# Association of obesity to reaction time and visual memory in schizophrenia

**DOI:** 10.1016/j.scog.2024.100316

**Published:** 2024-05-09

**Authors:** J.S. Toimela, A.H. Halt, M. Kerkelä, O. Kampman, J. Suvisaari, T. Kieseppä, M. Lähteenvuo, J. Tiihonen, A. Ahola-Olli, J. Veijola, M. Holm, The SUPER researchers listed in the Acknowledgements

**Affiliations:** a Research Unit of Clinical Medicine, University of Oulu, P.O. Box 5000, FI-90014 Oulu, Finland; b Department of Psychiatry, Oulu University Hospital, FI-90220 Oulu, Finland; c Mental Health Unit, Finnish Institute for Health and Welfare (THL), FI-00271 Helsinki, Finland; d University of Helsinki, Helsinki University Hospital, Psychiatry, FI-00029 Helsinki, Finland; e Department of Forensic Psychiatry, Niuvanniemi Hospital, University of Eastern Finland, FI-70240 Kuopio, Finland; f Department of Clinical Neuroscience, Karolinska Institutet, SE-17177 Stockholm, Sweden; g Center for Psychiatry Research, Stockholm City Council, SE-11364 Stockholm, Sweden; h Institute for Molecular Medicine Finland (FIMM), University of Helsinki, FI-00014 Helsinki, Finland; i Department of Internal Medicine, Satasairaala Hospital, Pori, Finland; j Department of Clinical Sciences, Psychiatry, Umeå University, Umeå SE-90187, Sweden; k University of Turku, Faculty of Medicine, Department of Clinical Medicine (Psychiatry), Turku, Finland; l The Wellbeing Services Country of Ostrobothnia, Department of Psychiatry, Vaasa, Finland; m The Pirkanmaa Wellbeing Services Country, Department of Psychiatry, Tampere, Finland; n Medical Research Center Oulu, Oulu University Hospital and University of Oulu, Oulu, Finland

**Keywords:** Schizophrenia, Schizoaffective disorder, Obesity, Cognition, Reaction time, Visual memory

## Abstract

**Background:**

Both overweight and cognitive deficits are common among people with schizophrenia (SZ) and schizoaffective disorder. The results in earlier studies have been inconsistent on whether overweight is associated with cognitive deficits in psychotic disorders.

**Aims:**

Our aim in this study was to detect possible associations between obesity and cognitive deficits among study participants with SZ and schizoaffective disorder.

**Methods:**

The study sample included 5382 participants with a clinical diagnosis of SZ or schizoaffective disorder selected from the Finnish SUPER study. Obesity was measured both with body-mass index and waist circumference. The cognitive performance was evaluated with two tests from the Cambridge automated neuropsychological test battery: Reaction time was evaluated with the 5-choice serial reaction time task. Visual memory was evaluated with the paired associative learning test. The final analysis included a total sample of 4498 participants applicable for the analysis of the reaction time and 3967 participants for the analysis of the visual memory.

**Results:**

Obesity measured with body-mass index was associated with better performance in reaction time task among both female and male participants. Among male participants, overweight was associated with better performance in the visual memory test. The waist circumference was not associated with cognitive measures.

**Conclusions:**

The results suggest that obesity in people with SZ or schizoaffective disorder might not be associated with cognitive deficits but instead with better cognitive performance. The results were opposite from earlier literature on the general population. More research is required to better understand whether the results might be partly caused by the differences in the etiology of obesity between the general population and people with SZ.

## 1 Introduction

Several trials have found an association between obesity and cognitive deficits (Fonds et al., 2006; Fagundo et al., 2012). A recent meta-analysis including 72 studies found obesity to be significantly associated with a broad range of executive function deficits (Yang et al., 2018). Another meta-analysis reported an association between obesity and almost all cognitive domains, including visual memory (Prickett et al., 2015). A systematic review found that the association between obesity and cognitive deficits was not present in all studies and that the association was more consistent to diabetes and hypertension (van den Berg et al., 2009). A large, recent meta-analysis presented that even though many studies have reported associations between obesity and cognitive deficits, the association is present only in the age above 65 (Tang et al., 2021). Another review article reviewed the relevant literature and presented that obesity could affect cognition via a variety of different mechanisms in the brain structures and functions (Wang et al., 2016).

Studies have shown that patients diagnosed with SZ are, compared with the general population, more often overweight and have other diseases related to obesity, for example, metabolic syndrome or cardiovascular disease. (De Hert et al., 2009). Their diet consists of more calories and their energy expenditures are lower than that of the general population (Manu et al., 2015). Additionally, several studies have found that patients with severe mental disorders, (e.g. SZ), are less physically active compared with the general population (Daumit et al., 2005, Chwastiak et al., 2011, Nyboe and Lund, 2013).

Deficits in cognition are common among people with SZ and cognitive deficits have also been discussed as a potential diagnostic criterion for SZ (Bora et al., 2010). Cognitive deficits in SZ comprise processing time, work memory, verbal learning and memory, visual learning and memory, reasoning, problem-solving, and verbal understanding (Nuechterlein et al., 2004).

Multiple studies have reported an association between obesity and cognitive deficits in SZ. For example, one study reported that obesity measured with the body-mass index (BMI^1^) was associated with lower results in multiple cognitive tests (Guo et al., 2013). Another study found that BMI was associated with impaired working memory and social cognition among SZ patients (Kimhy et al., 2014). A meta-analysis reported an association between obesity-related diseases (metabolic syndrome and diabetes) and cognitive deficiencies in SZ patients (Bora et al., 2017). In contrast, one study was not able to find an association between metabolic syndrome and cognition deficits among patients with SZ (Fang et al., 2019). In one study, an association was found between low BMI and high social cognition only among male patients with SZ (Cigliobianco et al., 2019).

A recent review suggested that waist circumference could be a better indicator of visceral adiposity and should be included when evaluating patients' obesity status (Ross et al., 2020). Waist circumference has also been used in previous studies focusing on cognition among patients with SZ. For example, high waist circumference has been associated with lower scores in attention among SZ patients (Lindenmayer et al., 2012). Similarly, another study found that waist circumference was negatively associated with cognitive functioning among patients with SZ (Storch Jakobsen et al., 2018).

There is still a lot of contradiction about the relationship between BMI and cognition in SZ. A recent systematic review and meta-analysis reported that, in contrast to metabolic syndrome, diabetes, and hypertension, obesity was not significantly associated with cognitive impairment (Hagi et al., 2021). For example, one study with a total sample of 417 SZ patients, found no association between BMI and cognitive performance (Depp et al., 2014). Another recent study conducted with Chinese SZ patients found that the obese participants presented a better cognitive performance compared with the normal weight patients (Tian et al., 2020).

Because of the discrepant results, more research is required to better understand the association between obesity and cognitive abilities in SZ. We were able to explore the relationship between obesity and cognition in a large sample of patients with SZ or schizoaffective disorders. To our best knowledge, there are no previous studies conducted directly on the association between obesity and the reactive and visual domains of cognition among patients with SZ.

The specific research question of the study is the following: Is there an association between overweight (measured with BMI or waist circumference) and reaction time or visual memory among people with SZ? Based on the previous literature we hypothesize that the reaction time is longer, and the visual memory is worse among overweight individuals compared with normal-weight individuals in the Finnish SZ population. Obesity (measured both by waist circumference and BMI) is expected to present an association with cognitive deficits and we expect to detect this association more clearly among male participants.

## 2 Material and methods

### 2.1 Study population

The participants of the present study were part of the SUPER-study focusing on psychotic illnesses in Finland. The SUPER-study is part of the international Stanley Global Neuropsychiatric Genomics Initiative, USA. The data for the SUPER-study was collected between the years 2016 and 2019. The participants in SUPER-study had a clinical diagnosis of schizophrenia spectrum psychotic disorder, bipolar disorder, or major depressive disorder with psychotic features. The voluntary participants were recruited from various inpatient and outpatient clinics, general health care centers, and supported housing as well as through newspaper advertisements. The study population was recruited from all levels of the healthcare system and in multiple geographic locations throughout Finland to enable inclusive sampling. The participants were interviewed and filled out a survey, as well as performed the chosen cognitive tests. All participants signed written informed consent.

A favorable statement for the SUPER-study was received from the Coordinating Ethics Committee of the Hospital District of Helsinki and Uusimaa. Following this, permission was gathered from all the healthcare organizations participating in this study. The SUPER research was executed by the following research ethics guidelines: The Responsible Conduct of Research and Procedures for Handling Allegations of Misconduct in Finland (http://www.tenk.fi/sites/tenk.fi/files/HTK_ohje_2012.pdf, accessed on 16th December 2022) and The European Code of Conduct for Research Integrity, revised edition 2017 (https://www.allea.org/wp-content/uploads/2017/05/ALLEA-European-Code-of-Conduct-for-Research-Integrity-2017.pdf, accessed on 16th December 2022).

**Fig. 1:**
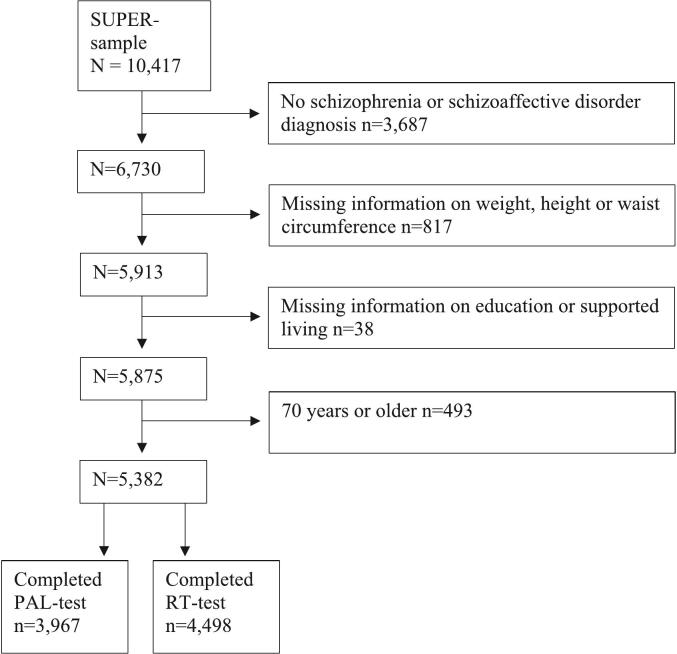
The flow chart of the SUPER study participants.

Out of the original SUPER participants (*n* = 10,417), we included in the present study the participants with a clinical diagnosis of schizophrenia or schizoaffective disorder (*n* = 6730). The participants with missing information on weight, height, or waist circumference information were removed (*n* = 817). After removing participants with missing education or supported housing information (*n* = 38) as well as participants older than 70 years (*n* = 493) the study sample included 5382 participants, with a total of 4498 participants performing reaction time task (study sample of reaction time task) and 3967 participants performing paired associative learning test (study sample of paired associative learning test, Fig. 1).

### 2.2 Schizophrenia diagnosis

The psychiatric diagnoses were drawn from the Care Register of Health Care. Finland uses the ICD-classification (International Statistical Classification of Diseases and Related Health Problems) for diagnosing psychotic disorders. ICD-8 was used in 1968–1986, ICD-9 in 1987–1995, and ICD-10 since 1996. This study included the diagnoses 295 (according to ICD-8 and ICD-9) and F20 (according to ICD-10) for SZ, as well as the diagnoses 295.7 (according to ICD-8 and ICD-9) and F25 (according to ICD-10) for the schizoaffective disorder.

### 2.3 Overweight classification

In this study, overweight was examined with BMI and waist circumference. The waist circumference as well as the height and weight of the participant were measured during the clinical examination. BMI was calculated by dividing the weight of the participant (in kilograms) by the square of their length (in meters). BMI > 25 has been considered to represent overweight (Huxley et al., 2010). In this study, we used the WHO BMI classification. According to the WHO, normal weight was between BMI 18.5–25 (https://www.who.int/europe/news-room/fact-sheets/item/a-healthy-lifestyle---who-recommendations, accessed on 16th December 2023). BMI between 25.0 and 30.0 was considered overweight, and BMI between 30.0–35.0, 35.0–40.0, and 40.0+ constituted obesity classes I, II, and III. Waist circumference has different margins for abdominal obesity for both females and males. We used the WHO waist circumference cut-off points for waist circumference classification (https://apps.who.int/iris/bitstream/handle/10665/44583/9789241501491_eng.pdf;jsessionid=5BAA3B0718CDA757C05CD83F393283EB?sequence=1, accessed on 16th December 2023). For females, waist circumference > 80 cm was considered as increased and >88 cm as substantially increased. For males increased waist circumference was >94 cm and >102 cm was substantially increased.

### 2.4 RT and PAL screening

In this study, the tests used for determining the cognitive performance of the participants have been chosen from The Cambridge neuropsychological test automated battery. The nurses were trained to supervise the tests and the tests were done during the daytime. The 5-choice serial reaction time task was performed to measure participants' reaction time (RT). In this task, the participant is asked to react to a series of light stimuli as fast as possible (Robbins, 2002). We used the median of the recorded reaction times as a variant. To examine the visual memory, participants performed the paired associative learning (PAL) test, in which the participant is required to learn visual patterns on a touch screen (Barnett et al., 2016). PAL TEA (total errors adjusted) was a dichotomized variable with a cutoff on the 50th percentile (total error scores at 10 or below) for good performance at the PAL test whereas the variable PAL FTMS (first time memory score) measures the performance on the first attempt on the test.

### 2.5 Covariates

We have chosen education, age, antipsychotic or benzodiazepine usage, supported housing, years from the first diagnosis of a psychosis, number of the hospitalizations and the smoking as the covariates for this study. The information on confounders was obtained through questionnaires and interviews during the clinical examination. Education has been associated with better cognitive performance (Ritchie and Tucker-Drob, 2018). The level of education was based on the interview data, where the education options were the following: basic level or no education, intermediate level, lowest level tertiary education, lower-degree level tertiary education, and upper-degree level tertiary education. In this study, low level education included basic level or no education, middle level included intermediate level and high level included the rest of the options. The usage of antipsychotics has been associated with deficits in various cognitive areas (Baldez et al., 2021). Benzodiazepines have been associated with cognitive impairment (Crowe and Stranks, 2018). The medication information was obtained from the interview. Information on supported housing was based on the interview data and was classified as supported (supported housing with full supervising/ supported housing without night supervising) or other (alone, with children without a spouse, with parents or siblings, with a spouse, or with spouse and children). The duration of the disease has been associated with cognitive impairment (Díaz-Caneja et al., 2015.). The number of the hospitalizations has been associated with the cognitive changes even though the evidence for the causation is uncertain (Harvey et al., 2013). The duration of the disease was calculated by subtracting the participants age of the first psychotic diagnosis given from the current age of examination. The smoking has been associated with cognitive impairment in many cognitive areas (Coustals et al., 2020). We included smoking as a binomial covariant. The first group included the patients with smoking during the day of examination or the previous day. The second group included all other patients.

### 2.6 The statistical methods

For each combination of the overweight (BMI or waist circumference), sex (female or male), and cognition variable (RT median, PAL TEA, or PAL FTMS) there were two different models created. The first models (age co-variated models) included only chosen obesity and cognitive variables along with age as a covariate. These models were created to see if obesity and the cognitive test results were associated when only the age was covaried. The second model class included all the covariates: age, education, supported housing, usage of antipsychotics or benzodiazepine, years from the first psychosis diagnosis, number of the hospitalizations and the smoking.

The RT median was analyzed with log-linear regression and the PAL-FTMS with linear regression. The PAL-TEA variable was analyzed with logistic regression. Within the RT analysis, lower reaction time score represented a better performance. Oppositely in the PAL analysis, a higher value represented better performance in the test. The RT test population (included all participants with graded RT scores) included a total sample of 2032 females and 2466 males. The test population included only 13 female and 12 male underweight participants with valid performance in the RT test and therefore the underweight participants have been excluded from the following analysis. The PAL test population (included all participants with graded PAL scores) included a total of 1785 females and 2182 males. Similarly, the PAL test population included a small number of underweight participants (11 females, 11 males), and they were also excluded from the following analysis.

## 3 Results

### 3.1 Descriptive statistics

Full data (participants with graded RT and/or PAL scores) included a total sample of 2386 females and 2996 males (Table 1). Female participants belonged most often to the obesity I -class (BMI = 30–34.5; 26.0 %) whereas the most common class in males was overweight (BMI = 25–29.9; 35.6 %). The majority of both the females and males belonged in the substantially increased class of the waist circumference classification (Females >88 cm: 83.1 %, males >102 cm: 64.6 %).

**Table 1:** The SUPER study participants by BMI and waist circumference classes separately in females and males.

	Female	Male
n	2386	2996
BMI class		
Underweight	19 (0.8 %)	20 (0.7 %)
Normal	384 (16,1 %)	670 (22.4 %)
Overweight	613 (25.7 %)	1068 (35.6 %)
Obesity I	620 (26.0 %)	750 (25.0 %)
Obesity II	370 (15.5 %)	345 (11.5 %)
Obesity III	380 (15.9 %)	143 (4.8 %)
Waist circumference class		
Normal (females <80 cm, males <94 cm)	182 (7.6 %)	531 (17.7 %)
Increased (females 80 cm–88 cm, males <94-102 cm)	222 (9.3 %)	531 (17.7 %)
Substantially increased (females >88, males >102 cm)	1982 (83.1 %)	1934 (64.6 %)

The mean age in the study population was 47 years among females and 46 years among males (Table 2). The majority of 87.4 % of females and 88.0 % of males were on antipsychotic medication. Additionally, 34.2 % of female participants and 29.5 % of male participants were on benzodiazepines. Females were less often living in supported housing than males (29.3 % of females compared to 39.0 % among males). 22.4 % of female participants had received a high education compared to the 13.6 % of males. 38.4 % of the female and 54.5 % of the male participants had smoked on the day of the examination or the previous day. The average duration of the psychotic illness was 20 years within both sexes. Most of the participants (87.7 % of females and 87.5 % of males) had been hospitalized more than once due to a psychosis.

**Table 2:** The background characteristics of the SUPER-study participants, separately in females and males.

	Females	Males
n	2386	2996
Supported housing	699 (29.3 %)	1168 (39.0 %)
Education		
High	534 (22.4 %)	407 (13.6 %)
Middle	1088 (45.6 %)	1376 (45.9 %)
Low	764 (32.0 %)	1213 (40.5 %)
Usage of any benzodiazepines	816 (34.2 %)	883 (29.5 %)
Usage of any antipsychotics	2085 (87.4 %)	2635 (88.0 %)
Age median	47 yrs.	46 yrs.
Smoking	885 (38.4 %)	1548 (54.5 %)
Years from the diagnosis	20 yrs.,	20 yrs.
Hospitalizations due to psychosis		
None	76 (3.2 %)	70 (2.3 %)
Once	217 (9.1 %)	304 (10.1 %)
More than once	2093 (87.7 %)	2622 (87.5 %)

### 3.2 Association between obesity and cognition

Females in the obesity II -class had lower RT-scores (*p* = 0.030) representing better performance in the reaction time task compared to the females with normal weight (Table 3). Similarly, males in the overweight-class had better performance in reaction time task (*p* = 0.049) compared to the males with normal weight. Additionally among males, participants in overweight (*p* = 0.024) and obesity I (*p* = 0.015) classes had better performance in the paired learning test only when measured with PAL FTMS compared to the normal weight males. Waist circumference was not associated with cognitive measures among female or male participants (Table 4).

**Table 3:** Association between BMI classes and cognitive factors with female and male participants (normal weight class as reference).

	Overweight^a^		Obesity class I^b^		Obesity class II^c^		Obesity class III^d^	
Females								
**RT Median**^e^	**e**^**β**^**(95** **% CI)**	**p-value**	**e**^**β**^**(95** **% CI)**	**p-value**	**e**^**β**^**(95** **% CI)**	**p-value**	**e**^**β**^**(95** **% CI)**	**p-value**
Crude^h^	0.99 (0.97–1.02)	0.649	0.98 (0.96–1.01)	0.216	0.97 (0.95–1.00)	0.077	0.99 (0.97–1.02)	0.667
Adjusted^i^	0.99 (0.97–1.02)	0.625	0.98 (0.96–1.01)	0.130	0.97 (0.94–1.00)	0.032	0.99 (0.96–1.02)	0.461
**PAL FTMS**^f^	**β (95** **% CI)**	**p-value**	**β (95** **% CI)**	**p-value**	**β (95** **% CI)**	**p-value**	**β (95** **% CI)**	**p-value**
Crude^h^	−0.52 (−1.19–0.15)	0.129	−0.39 (−1.06–0.28)	0.254	−0.36 (−1.12–0.39)	0.203	−0.48 (−1.21–0.26)	0.129
Adjusted^i^	−0.22 (−0.86–0.43)	0.508	0.02 (−0.63–0.66)	0.957	0.15 (−0.59–0.89)	0.694	−0.01 (−0.73–0.71)	0.977
**PAL TEA**^g^	**OR (95** **% CI)**	**p-value**	**OR (95** **% CI)**	**p-value**	**OR (95** **% CI)**	**p-value**	**OR (95** **% CI)**	**p-value**
Crude^h^	0.63 (0.43–0.93)	0.020	0.73 (0.50–1.08)	0.111	0.62 (0.40–0.96)	0.035	0.57 (0.37–0.86)	0.009
Adjusted^i^	0.72 (0.48–1.08)	0.108	0.93 (0.62–1.39)	0.711	0.83 (0.51–1.32)	0.430	0.74 (0.47–1.17)	0.204

Males								
**RT Median**^e^	**e**^**β**^**(95** **% CI)**	**p-value**	**e**^**β**^**(95** **% CI)**	**p-value**	**e**^**β**^**(95** **% CI)**	**p-value**	**e**^**β**^**(95** **% CI)**	**p-value**
Crude^h^	0.98 (0.96–0.99)	0.009	0.97 (0.95–0.99)	0.009	0.98 (0.95–1.00)	0.051	0.97 (0.94–1.00)	0.060
Adjusted^i^	0.98 (0.96–1.00)	0.092	0.99 (0.97–1.01)	0.208	0.99 (0.96–1.01)	0.353	0.98 (0.95–1.02)	0.368
**PAL FTMS**^f^	**β (95** **% CI)**	**p-value**	**β (95** **% CI)**	**p-value**	**β (95** **% CI)**	**p-value**	**β (95** **% CI)**	**p-value**
Crude^h^	0.77 (0.26–1.28)	0.003	0.90 (0.35–1.44)	0.001	0.87 (0.19–1.55)	0.012	0.50 (−0.39–1.39)	0.269
Adjusted^i^	0.54 (0.03–1.04)	0.039	0.52 (−0.03–1.07)	0.062	0.67 (−0.01–1.35)	0.052	0.43 (−0.48–1.33)	0.356
**PAL TEA**^g^	**OR (95** **% CI)**	**p-value**	**OR (95** **% CI)**	**p-value**	**OR (95** **% CI)**	**p-value**	**OR (95** **% CI)**	**p-value**
Crude^a^	1.32 (0.93–1.90)	0.121	1.30 (0.89–1.91)	1.180	1.00 (0.60–1.53)	0.999	1.08(0.57–1.97)	0.801
Adjusted^b^	1.30 (0.90–1.89)	0.169	1.26 (0.85–1.90)	0.254	1.04 (0.62–1.72)	0.878	1.20 (0.62–2.21)	0.575

a Overweight: BMI 25.0–30.0.

b Obesity class I: BMI 30.0–35.0.

c Obesity class II: BMI 35.0–40.0.

d Obesity class. III: BMI > 40.0.

e RT Median = reaction time median score.

f PAL FTMS = paired associative learning - first time memory score.

g PAL TEA = paired associative learning – total errors adjusted.

h Adjusted with age.

i Adjusted with age, supported living, education and the usage of antipsychotic medication or benzodiazepines.

**Table 4:** Association between waist circumference and cognitive measures with female and male participants (normal weight class as reference).

	Increased^a^		Substantially increased^b^	
Females				
**RT Median**^c^	**e**^**β**^**(95** **% CI)**	**p-value**	**e**^**β**^**(95** **% CI)**	**p-value**
Crude^f^	1.00 (0.96–1.03)	0.804	1.01 (0.98–1.04)	0.508
Adjusted^g^	0.99 (0.95–1.03)	0.666	1.00 (0.97–1.03)	0.851
**PAL FTMS**^d^	**β (95** **% CI)**	**p-value**	**β (95** **% CI)**	**p-value**
Crude^f^	−0.15 (−1.16–0.86)	0.772	−0.91 (−1.69–(−0.12))	0.024
Adjusted^g^	−0.04 (−1.00–0.93)	0.939	−0.27 (−1.04–0.49)	0.484
**PAL TEA**^e^	**OR (95** **% CI)**	**p-value**	**OR (95** **% CI)**	**p-value**
Crude^f^	1.06(0.63–1.79)	0.818	0.65(0.44–0.99)	0.040
Adjusted^g^	1.10 (0.64–1.90)	0.720	0.88 (0.57–1.36)	0.546

Males				
**RT Median**^c^	**e**^**β**^**(95** **% CI)**	**p-value**	**e**^**β**^**(95** **% CI)**	**p-value**
Crude^f^	0.98 (0.96–1.00)	0.092	0.98 (0.96–1.00)	0.073
Adjusted^g^	0.98 (0.96–1.00)	0.098	0.99 (0.97–1.01)	0.325
**PAL FTMS**^d^	**β (95** **% CI)**	**p-value**	**β (95** **% CI)**	**p-value**
Crude^f^	0.29 (−0.34–0.92)	0.374	0.31 (−0.20–0.81)	0.238
Adjusted^g^	0.36 (−0.27–0.98)	0.266	0.29 (−0.22–0.80)	0.266
**PAL TEA**^e^	**OR (95** **% CI)**	**p-value**	**OR (95** **% CI)**	**p-value**
Crude^f^	0.93 (0.61–1.43)	0.753	1.00 (0.71–1.42)	0.986
Adjusted^g^	0.89 (0.59–1.34)	0.592	0.94 (0.68–1.30)	0.694

a Increased waist circumference: females 80–88 cm, males 94–102 cm.

b Substantially increased waist circumference: females >88 cm, males >102 cm.

c RT Median = reaction time median score.

d PAL FTMS = paired associative learning - first time memory score.

e PAL TEA = paired associative learning – total errors adjusted.

f Adjusted with age.

g Adjusted with age, supported living, education and the usage of antipsychotic medication or benzodiazepines.

## 4 Discussion

This study aimed to explore the potential association between obesity and cognitive deficits in reaction time and visual memory among people with SZ or schizoaffective disorder. The study sample was large, including a total of 5382 participants. The results did not support our hypothesis that overweight would be positively associated with reaction time, and negatively associated with visual memory. In our study, obese female participants were associated with better performance in the reaction time task compared with the normal-weight participants. No association was present between obesity and visual memory among female participants. Male participants classified as overweight performed better in the reaction time task compared with normal-weight male participants. Obese male participants performed also better in the visual memory test. In our study the *p*-values were borderline significant which is a common problem in similar studies with large study populations.

As priorly addressed, the results on the association between obesity and cognitive performance among SZ patients have remained incoherent throughout the previous literature. For the support of our findings, another recent study has also found an association between BMI and enhanced cognitive performance in language and visuospatial/construction domains among patients with SZ (Wei et al., 2020). Additionally, our findings support the findings of Tian et al., 2020, which suggest that obesity might be associated with enhanced cognitive performance. One potential explanation for this is that the usage of antipsychotics among patients with SZ might be associated with a more nutrient-rich diet causing better brain function (Tian et al., 2020).

We suspect that our results might be partly caused by the differences in the etiology of obesity between the general population and people with SZ. The factors causing obesity vary between these groups: Within the general population, the obesogenic environment including a sedentary lifestyle, eating habits, and urbanization have been presented as some of the leading causes of obesity (Endalifer and Diress, 2020). Among patients with schizophrenia, the development of obesity is often associated with different factors, especially with medication. Both conventional and newer antipsychotics have been associated with significant weight gain (Allison et al., 1999). Out of the newer antipsychotics, olanzapine is associated with the most weight gain. Weight gain is also a common side effect of antidepressant usage (Alonso-Pedrero et al., 2019).

It has been presented that, in the general population, most of the obese children remain obese in adulthood (Simmonds et al., 2016). We suspect that patients with SZ might be better protected from the obesity-associated cognitive deficiencies compared with the general population. This might be due to the common incidence of obesity after the introduction of antipsychotics and antidepressants later in life.

Previously it has been presented that the distribution of the adipose tissue can alter the risk for cognitive deficits in the general population. For example, visceral obesity is associated with greater cognitive deficits compared to peripherally distributed adipose tissue (Tanaka et al., 2020). It has also been shown that the distribution of adipose tissue differs between females and males and that males are more prone to gain visceral fat (Palmer and Clegg, 2015). These findings suggest that viscerally obese males would be especially subject to enhanced cognitive decline. As addressed in the introduction, waist circumference can be used as a measurement for visceral obesity. Parallel to most of the prior studies, visceral obesity was not associated with better cognitive performance in our study.

### 4.1 Strengths and limitations

In this study, we were able to use a larger study population compared to most of the previous studies in this field. The study sample was nationwide, and the data was collected in multiple healthcare centers. Age, education, living style, the usage of antipsychotics or benzodiazepines, years from the first psychosis diagnosis, number of the hospitalizations and the smoking were chosen as the covariates in the statistical analysis. In this study we were only able to use two measurements from the CANTAB cognitive test battery. A wider test battery could have been beneficial. The usage of antipsychotics is heavily associated with weight gain (Barton et al., 2020).

### 4.2 Conclusions

Our results did not support our hypothesis that obese male participants would have lower cognitive functioning when measured with the chosen cognitive factors. On the contradictory, obesity could be associated with better cognitive performance, especially among male participants. Interestingly, obesity was not associated with enhanced cognition when obesity was measured with waist circumference. This indicates, similarly to prior literature, that visceral obesity is not associated with better cognitive performance. Additionally, the results were also partly contrary to those reported among the general population. This might be partly caused by the differences in the etiology of obesity between the general population and people with SZ. More research is required to better understand this association.

## Role of the funding source

The authors received no financial support for the research, authorship or publication of this article.

## CRediT authorship contribution statement

**J.S. Toimela:** Conceptualization, Writing – original draft, Writing – review & editing. **A.H. Halt:** Conceptualization, Project administration, Supervision, Writing – original draft, Writing – review & editing. **M. Kerkelä:** Formal analysis, Methodology. **O. Kampman:** Data curation, Supervision. **J. Suvisaari:** Data curation, Supervision. **T. Kieseppä:** Data curation, Supervision. **M. Lähteenvuo:** Data curation, Supervision. **J. Tiihonen:** Data curation, Supervision. **A. Ahola-Olli:** Data curation, Supervision. **J. Veijola:** Conceptualization, Data curation, Methodology, Project administration, Supervision, Writing – original draft, Writing – review & editing. **M. Holm:** Methodology, Supervision, Writing – original draft, Writing – review & editing.

## Declaration of competing interest

Dr. Tiihonen has participated in research projects funded by grants from Janssen-Cilag and Eli Lilly to his employing institution. Dr. Tiihonen reports personal fees from Eli Lilly, Evidera, HLS Therapeutics, Janssen-Cilag, Lundbeck, Orion, Otsuka, Mediuutiset, Sidera, and Sunovion. The other authors declare no conflict of interest.

## Footnotes

1 BMI = body-mass index
